# BTLA-HVEM Couple in Health and Diseases: Insights for Immunotherapy in Lung Cancer

**DOI:** 10.3389/fonc.2021.682007

**Published:** 2021-08-31

**Authors:** Clemence Demerlé, Laurent Gorvel, Daniel Olive

**Affiliations:** Cancer Research Center in Marseille (CRCM), INSERM U1068, CNRS U7258, Aix Marseille University (AMU), Paoli Calmette Institute (IPC), Marseille, France

**Keywords:** immune escape, lung cancer, T cell exhaustion, HVEM/TNFRSF14, BTLA, immunotherapy

## Abstract

Lung cancer is the leading cause of cancer deaths worldwide. Immunotherapies (IT) have been rapidly approved for lung cancer treatment after the spectacular results in melanoma. Responses to the currently used checkpoint inhibitors are strikingly good especially in metastatic diseases. However, durable responses are observed in only 25% of cases. Consequently, there is an urgent need for new immunotherapy targets. Among the multiple checkpoints involved in the tumor immune escape, the BTLA-HVEM couple appears to be a promising target. BTLA (B- and T- Lymphocyte Attenuator) is a co-inhibitory receptor mainly expressed by B and T cells, repressing the activation signal transduction. BTLA shares similarities with other immune checkpoints such as PD-1 and CTLA-4 which are the targets of the currently used immunotherapies. Furthermore, BTLA expression points out terminally exhausted and dysfunctional lymphocytes, and correlates with lung cancer progression. The ligand of BTLA is HVEM (Herpes Virus Entry Mediator) which belongs to the TNF receptor family. Often described as a molecular switch, HVEM is constitutively expressed by many cells, including cells from tumor and healthy tissues. In addition, HVEM seems to be involved in tumor immuno-evasion, especially in lung tumors lacking PD-L1 expression. Here, we propose to review the role of BTLA-HVEM in immuno-escape in order to highlight its potential for designing new immunotherapies.

## Introduction 

For decades, tumors have been directly targeted by chemotherapy, radiotherapy, or resected when possible. The association of these treatments often leads to tumor eradication. However, collateral effects on the non-tumor cells are not negligible. Mesenchymal and immune cells in the tumor environment are either resident or recruited, and promote or inhibit tumor growth. On the one hand, the infiltration of the tumor by immunosuppressive cells such as regulatory T cells (Tregs), myeloid derived suppressor cells (MDSCs), and tumor associated macrophages (TAM) is prejudicial to tumor immune control and lead to an unfavourable prognosis (1). On the other hand, M1 macrophages, Natural Killer cells, T CD8^+^, and T γδ lymphocytes are crucial for anti-tumor immunity. In addition to divert key resources from their environment, tumor cells developed mechanisms to evade immune recognition and switch cytotoxic cells off (2). Among these mechanisms, one of the most studied is lymphocytes exhaustion through co-inhibitory molecules signalling. Co-inhibitory and co-stimulating receptors expressed by T lymphocytes are known as immune checkpoints (3). The balance between the signals received through these receptors determines lymphocytes activation. Tumors are able to escape immune response by reducing the expression of costimulatory ligands or upregulating the co-inhibitory molecules. For example, the co-inhibitory receptor Programmed cell Death 1 (PD-1) and its ligands PD-L1 and PD-L2 are often overexpressed by tumors to inhibit T cells activation (4).

Immunotherapies (IT) have changed the paradigm in cancer treatments. Instead of the direct tumor killing by chemotherapy or radiation, IT acts on immune cells to turn them into *in situ* weapons to eliminate tumor cells. Current ITs are antagonistic antibodies, which block co-inhibitory signalling such as the CTLA-4/CD80-CD86 and PD-1/PD-L1-PD-L2 pathways. After the promising results in metastatic melanoma, anti-PD-1 IT (Pembrolizumab and Nivolumab) were rapidly approved for lung cancer treatment 6 years ago (5). Recently, anti-PD-L1 IT (Atezoliumab and Durvalumab) were also approved in this context. Anti-CTLA-4 efficiency remains unclear in lung cancer. FDA approval was only given in 2020 in combination with anti-PD-1 for metastatic NSCLC expressing PD-L1 (6). Biomarkers were studied to predict responses, including CD8^+^ T lymphocyte infiltration, PD-L1 tumor expression, or tumor mutational burden at diagnosis (7). These indicators about the local immune context remain weakly reliable to predict patient response to IT or who will suffer from hyper-progression (8). Altogether, current ITs for NSCLC show a long-term efficiency in 20%–30% of treated patients. Therefore, new IT strategies are needed to propose alternative treatments for advanced lung cancer patients who are not responding or are relapsing under anti-PD-1/PD-L1 IT.

BTLA (B and T Lymphocyte Attenuator) is another important co-inhibitory receptor which ligand is HVEM (Herpes Virus Entry Mediator). Although BTLA shares similarities with PD-1 and CTLA-4, they differ in terms of expression and functions. HVEM is widely expressed among cell types and participate to immune homeostasis. In tumors, HVEM upregulation was largely reported (**Table 1**). Here, we propose to review the implication of BTLA-HVEM in tumor immune-evasion and its potential for developing new IT to treat lung cancer.

**Table 1:** Review of HVEM upregulation in solid cancers.

Cancer	Number of patients	HVEM positivity	PD-L1 status	Disease Progression	Prognosis	Year of publication	Reference
Melanoma	116	98.3%	Mainly Mutual exclusive	n.a	bad	2019	(9)
Colorectal cancer	234	94,9%	n.a	more advanced tumor status and pathological stage	bad	2015	(10)
Gastric cancer	136	89.0%	n.a	Lymph node metastasis and depth of invasion	bad	2017	(11)
Glioblastoma	34	72.7%	n.a	n.a	bad	2019	(12)
hepatocellular carcinoma	150	Only low/high HVEM status	n.a	Intra and extra hepatic recurrences	bad	2015	(13)
clear cell renal carcinoma	140	Only low/high HVEM status	n.a	n.a	bad	2019	(14)
human oesophageal squamous cell carcinoma	103	Only low/high HVEM status	n.a	Depth of tumor invasion and lymph node metastasis	bad	2013	(15)
breast cancer	1005	16.5%	Negative correlation	High grade and advanced pathological stage	bad	2017	(16)
Non-small cell-lung cancer	527	18.6%	Negative correlation	lymph node N2 metastasis	Not significant	2018	(17)

This table reviews the published studies on HVEM expression and correlation with disease progression and prognosis. (n.a, not assessed).

## BTLA and HVEM Implication in Immune Homeostasis

### BTLA

B and T cell attenuator (BTLA) was discovered after PD-1 and CTLA-4 almost 20 years ago (18). BTLA belongs to the CD28 family and shares structural similarities with PD-1 and CTLA-4. It exhibits an extracellular immunoglobulin domain, an immunoreceptor tyrosine inhibitory motif (ITIM) as well as an immunoreceptor tyrosine-based switch motif (ITSM). BTLA signal transduction consists in the phosphorylation of ITIMs and Src homology 2 (SH2) domain–containing phosphatase 1 (SHP-1)/SHP-2 association, which leads to the repression of T cell proliferation and cytokine production (19). The inhibitory function of BTLA was confirmed in mice through a BTLA deficient model showing an enhanced sensitivity to auto-immune encephalomyelitis (18). *In vitro*, BTLA deficient T cells show an increased TCR-induced proliferation compared to normal T cells. In healthy humans, BTLA expression is high on naïve CD4^+^ and T CD8^+^ T cells from peripheral blood. BTLA expression remains high during CD4^+^ differentiation whereas BTLA is downregulated during CD8^+^ T cell differentiation (20). Similar results were observed on γδ-T cells, which is another cytotoxic population (21). BTLA is highly expressed on resting Vγ9Vδ2 cells, the major γδ-T-cell subset in human peripheral blood, and is downregulated during Vγ9Vδ2 differentiation. Serriari et al. (20) showed that BTLA expression is increased on CMV-specific CD8^+^ T cells and decreased on memory CD8^+^ T cell subsets when CMV infection is controlled. Furthermore, the authors demonstrated that *in vitro* BTLA blockade enhanced CD8^+^ T cell proliferation, suggesting that BTLA is a promising target to improve the control of viral infections.

Originally described on B and T lymphocytes, BTLA expression was more recently observed on murine type 1 conventional dendritic cells (cDC1) (22). BTLA-positive DCs take part in peripheral Treg induction in an acute encephalomyelitis mouse model. This beneficial tolerance mechanism was not observed with BTLA^-^ DCs. Zhang et al. reported that BTLA^+^DCs promoted Treg and Th2 polarization of T cells in human lung tuberculosis (23). Altogether, these data highlight the importance of BTLA in immune homeostasis.

### HVEM

HVEM, or TNFRSF14, is a TNF-receptor family member. It was discovered in 1996 for its role in the entry of Herpes Simplex Virus (HSV) into cells (24). HVEM expression is observed in tissues, with a higher expression in the lung, kidney, and liver, moderate expression in the heart, placenta, skeletal muscles, and pancreas, and very low in the brain. Among immune cells, HVEM is strongly expressed by resting T and B cells, NK cells, Tregs, monocytes, and DCs (25). Mesenchymal cells and epithelial cells also express HVEM (26). In epithelial cells, HVEM expression plays a critical role in innate mucosal defence against pathogenic bacteria (27). In a mouse model of acute enteropathogenic infection, authors found that HVEM^-/-^ mice present a severe infection with an increased inflammation, bacterial dissemination, and reduced survival. Similar results were found in another mice model of lung infection by *Streptococcus pneumoniae*, showing that epithelial innate immunity is impaired in HVEM^-/-^ condition.

**Figure 1:**
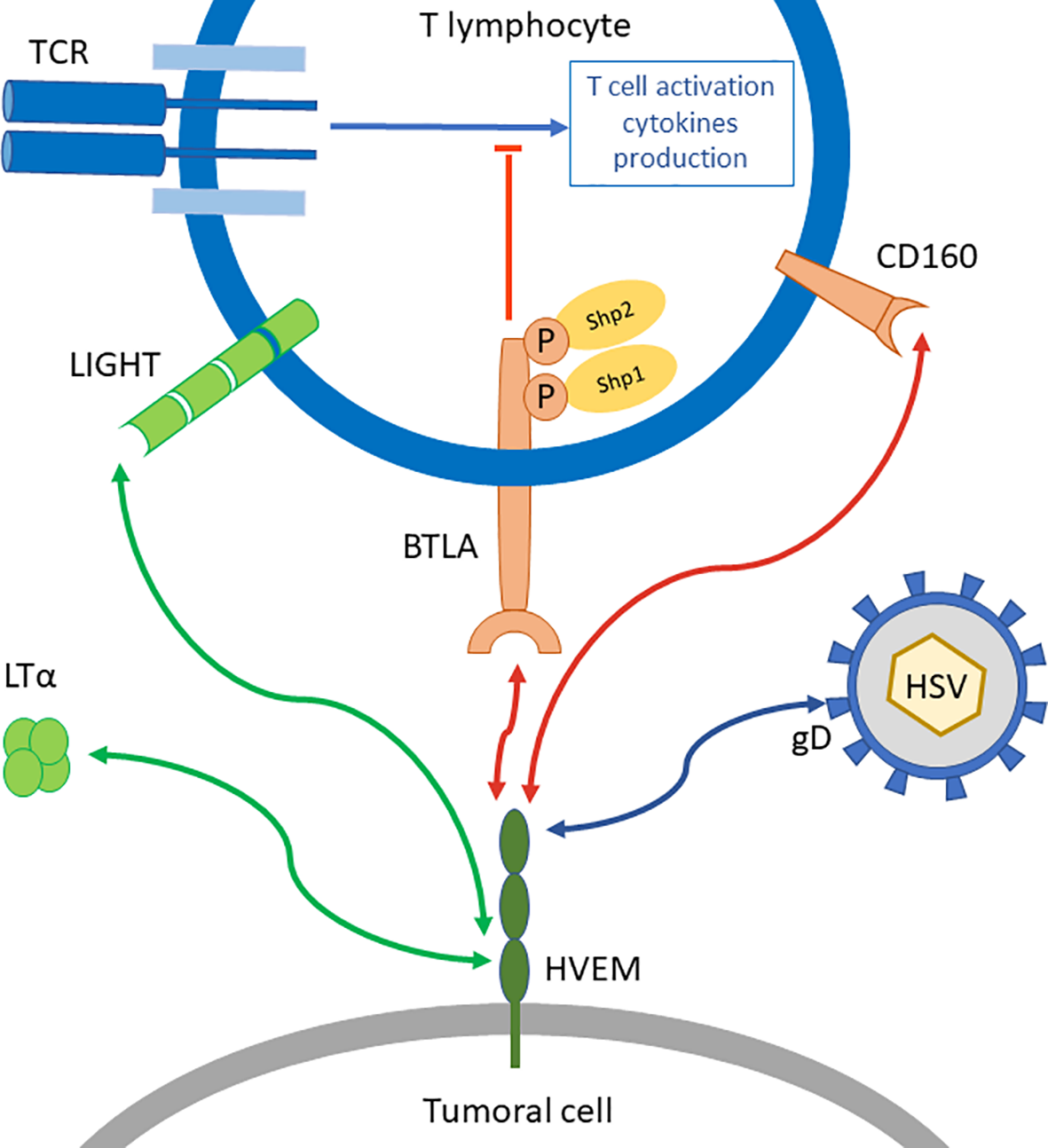
BTLA/HVEM network. BTLA is a co-inhibitory receptor that represses TCR signal transduction in T lymphocytes through the recruitment of Shp1 and Shp2. Its ligand HVEM can also bind other molecules such as LIGHT or Lymphotoxin-α (LTα) which triggers co-stimulatory signals (green arrows), or CD160 another co-inhibitory receptor (red arrows) on distinct binding sites. HVEM is also binding to glycoprotein D from Herpes Simplex Virus (HSV).

HVEM interaction with BTLA was the first ligation between an Ig structure and TNFR family to be described (28). HVEM has other ligands outside of BTLA (**Figure 1**). Indeed, HVEM binds to CD160, LIGHT, Lymphotoxin-α (LTα), and herpes simplex virus glycoprotein D. When HVEM is engaged with LIGHT or LTα, a co-stimulatory signal is delivered, while HVEM binding to BTLA or CD160 triggers a co-inhibitory signal. Thus, HVEM is often described as a molecular switch depending on the engaged ligand (29). However, HVEM^-/-^ mice show increased responses to T cell stimulation and enhanced susceptibility to auto-immune disorders (30). This suggests that HVEM co-inhibitory signal overcomes its HVEM co-stimulation counterpart.

### HVEM-BTLA Binding

BTLA-HVEM binding sites were investigated by crystallography. Compared to other CD28 receptors such as PD-1 and CTLA-4, BTLA differs by its extracellular domain lacking a C’’ strand. The crystal structure of BTLA-HVEM interaction indicates that BTLA binds to a N-terminal cysteine-rich domain of HVEM (31). Moreover, this cysteine-rich domain (CRD1) is also the binding site for CD160, the other inhibitory ligand of HVEM, but not for the costimulatory ligand LIGHT (32). Therefore, the inhibitory and stimulatory signals are received by HVEM on distinct structural binding sites. This suggests that inhibitory signals could be blocked without altering the binding of stimulatory ligands. These important structural aspects highlight the importance of modulation rather than complete inhibition of HVEM, which has a complex molecular network.

## HVEM Role in Tumor

### HVEM Dysregulation in Tumors

HVEM upregulation represents another immune escape mechanism, which is similar to the overexpression of PD-L1 or PD-L2 by tumor cells to engage PD-1 on immune cells. In melanoma, HVEM was shown to be overexpressed in some tumors and contiguous to BTLA positive T cells (9). Malissen et al. identified an “HVEM signature” gathering genes involved in melanoma proliferation and aggressiveness. Surprisingly, this signature was not correlated with a classical IFN-γ signature. HVEM expression was not linked to PD-L1 expression status. Moreover, HVEM high expression in their cohort was associated to a significantly poorer prognosis, which was confirmed in The Cancer Genome Atlas (TCGA) melanoma cohort.

In colorectal cancer, HVEM expression was upregulated in malignant lesions (10). High HVEM expression was associated to the tumor status and pathological stage. HVEM status was an independent prognostic value. In another study on 136 gastric cancer biopsies, an increased HVEM expression was associated to disease progression and poorer overall survival (11).

Interestingly, a recent study in glioblastoma highlighted similar results. HVEM expression was increased in aggressive subtypes of glioma, and was associated to a poorer prognosis (12). Assessed by IHC on 34 glioma tissues, HVEM expression was localized to the peri-necrotic zone and areas of microvascular proliferation. Based on transcriptomic analysis, HVEM expression was related to immune cell infiltration and stromal cells of the microenvironment. HVEM expression was also linked to the expression of PD-1, PD-L1, CTLA-4, LAG3, and VISTA, leading to the conclusion that HVEM is crucially involved in the modulation of the immune and inflammatory responses, especially T cell activation.

The negative prognosis impact of HVEM high expression was described in many others cancers such as hepatocellular carcinoma (13), clear cell renal carcinoma (14), human esophageal squamous cell carcinoma (15), and breast cancer (16) (**Table 1**). In the field of hematological malignancies, a higher HVEM expression in follicular lymphoma (33) and chronic lymphocytic leukemia (34) was correlated to a poorer prognosis.

Thus, the numerous data in hematological and solid tumors concerning HVEM role in tumor immune escape provide a strong rationale for the study of HVEM in lung cancer and assess its potential as a major candidate for IT.

### HVEM Dysregulation in Lung Cancer

In 2018, Ren et al. (17) published the first study dedicated to HVEM in lung cancer. They analysed the HVEM expression in 415 NSCLC biopsies and 56 NSCLC cell lines. HVEM expression was positive in 18.6% (77/415) of the biopsies and 48.2% (27/56) of the cell lines. PD-L1 expression was also evaluated by IHC in 491/527 patients, and 31% (152/491) were positive.

First, the authors showed that a higher HVEM expression was significantly associated with lymph node (N2) metastasis. A higher HVEM expression was also evidenced in the advanced stage group (stages III–IV) without reaching statistical significance. However, HVEM expression was not predictive of the overall survival. Noteworthy, HVEM status was not linked to age, gender, smoking status, oncogenic status, pathology, and ethnicity. Thus, the upregulation of HVEM would be a tumor-driven mechanism of immune escape that occurs during tumor growth and disease progression. Second, they found a negative correlation between PD-L1 and HVEM expression suggesting that the underlying mechanisms involved in PD-L1 or HVEM upregulation are different. So far, this is the first and only study which demonstrates the importance of HVEM in immune evasion in lung cancer, especially when PD-L1 is lacking.

Altogether, these data show that HVEM upregulation is closely linked to tumor progression and aggressiveness in many solid cancers, including lung cancer, and hematological malignancies.

## BTLA Role in Tumors

### BTLA Expression in Tumors

In melanoma, HVEM^+^ tumor cells were found to be contiguous to BTLA^+^ tumor infiltrating lymphocytes (TILs) (9). Gertner-Dardenne et al. showed that HVEM-positive lymphoma cells in contact with γδ- T cells polarize the distribution of BTLA and Vδ2-TCR to the immunological synapse (21). In gastric cancer, Lan et al. (11) reported an upregulation of HVEM in malignant lesion as discussed previously, but they also evaluated BTLA expression in the same biopsies. As HVEM, BTLA is more expressed in malignant tissues compared to normal tissues. Indeed, a higher BTLA expression was positively correlated with a higher HVEM expression. Moreover, BTLA expression impacted the prognosis, with a 5-year overall survival (OS) rate at 48.3% for the low BTLA expression group, falling to 17.9% when BTLA was highly expressed. A higher BTLA expression was also associated to lymph node metastasis.

BTLA is downregulated during physiological or virally-induced T cells differentiation (20, 21, 35), whereas in tumor conditions, BTLA expression follows a different pattern. Derré et al. (35) analyzed PBMC from melanoma patients, and showed that BTLA expression remains high on Melan-A^MART-1^–specific lymphocytes despite effector cell differentiation. This phenotype was reversed by conventional vaccination with Melan-A^MART-1^ peptide, which leads to a progressive BTLA downregulation on vaccine-specific CD8^+^ T cells. Furthermore, IFN-γ production is also restored showing that BTLA-triggered inhibition can be overcome.

BTLA is related to other co-inhibitory receptors. For this reason, BTLA was analyzed along with other co-inhibitory receptor expression. In advanced melanoma, Fourcade et al. (36) demonstrated that 42% of NY-ESO-1-specific CD8^+^ T lymphocytes co-expressed BTLA and PD-1. These cells have a partial dysfunctional phenotype compared to the PD1^+^ BTLA^+^ TIM-3^+^ subset, which is reported as a highly dysfunctional subset. TIM-3 and PD-1 are upregulated when NY-ESO-1-specific CD8^+^ T lymphocytes received a prolonged stimulation with a cognate antigen. BTLA expression followed a different pattern. This suggests that BTLA upregulation depends on different conditions, rather than a functional exhaustion driven by a high antigen load. Also, BTLA blockade by anti-BTLA antibody enhanced IFN-γ, TNFα, and IL-2 production by NY-ESO-1-specific CD8^+^ T cells. Interestingly, a synergistic effect was observed in functional assays when anti-BTLA was combined with anti-PD-1 IT.

### BTLA: The Keystone of T Cell Exhaustion in Lung Cancer

In lung cancer, BTLA expression and function is understudied. However, some critical data have been reported. Indeed, in a mouse model of subcutaneous lung tumor implantation, Mittal et al. showed that BTLA frequency on CD4^+^ and CD8^+^ T cells was increased, along with other co-inhibitory markers such as PD-1 and 2B4 (37). They concluded that T cell exhaustion was driven by the tumor implantation and modification of the TME. This study was confirmed and extended in the human lung tumor samples by Thommen et al. (38). Indeed, Thommen et al. described a progressive increase of dysfunctional T cells correlated to the expression of multiple co-inhibitory receptors and also to disease progression. Authors performed a detailed immunophenotyping of TILs from NSCLC biopsies (n = 25). They found that the expression of PD-1 and Tim-3 was increased on the infiltrating CD8^+^ T cell subset in advanced tumor stages. The percentage of BTLA positive cells was rather low, but following the same pattern as PD-1 and TIM-3, without reaching statistical significance. BTLA^+^ CD8^+^ T cells also highly expressed other co-inhibitory receptors, suggesting that BTLA was upregulated during the late stages of T cell exhaustion. According to the authors, the sequential expression of co-inhibitory receptors started from PD-1 expression, then TIM-3, CTLA-4, and LAG-3, and finally, BTLA. To summarize the expression of all co-inhibitory receptors, the authors designed an inhibitory receptor (IR) scoring system. The IR score increased in patients with lymph node invasion and advanced tumor stages. However, it did not correlate with the primary tumor size. Therefore, these data demonstrate that CD8^+^ T cell exhaustion evolves during tumor progression. To measure T cell dysfunction, the authors set an *in vitro* assay based on CD3/CD28 T cells activation with CD25 induction, granzyme-B expression, and cytokine production readouts. Results were rather heterogeneous among patients, ranging from highly dysfunctional state, which corresponds to a weak response after polyclonal activation, to lowly affected CD8^+^ T cells, which strongly respond to stimulation. Interestingly, the dysfunctional state was positively correlated with the IR score. Finally, authors demonstrated that PD-1 blockade can partially rescue T cell function depending on the PD-1 expression. In fact, only CD8^+^ T cells with an intermediate PD-1 expression benefit from the anti-PD-1 treatment to restore their function. This suggests that PD-1^high^ CD8^+^ T cells are too exhausted for their function to be restored by PD-1 blockade alone. To note, the PD-1^high^ subset also expresses higher amounts of TIM-3, CTLA-4, LAG-3, and BTLA compared to the PD-1^int^ subset. Thus, a combined strategy to complete PD-1 blockade is an interesting strategy to explore. So far, this major publication studying BTLA involvement in human lung cancer raises new insights for T cells dysfunction and tumor progression.

Noteworthy, Lou et al. investigated the correlation between epithelial-mesenchymal transition (EMT) and immune activation in lung cancer (39). They established that EMT was closely linked to an inflammatory transcriptomic signature. Moreover, in tumors displaying EMT, the transcriptomic expression of immune checkpoints, including BTLA, was increased and associated with regulatory T cells recruitment. Precisely, BTLA was found to be increased in mesenchymal tissue, suggesting that EMT could be modulated by the inflammatory micro-environment through a BTLA-dependent mechanism. This study opens a new field of research exploring BTLA expression on non-immune cells.

### BTLA Expression on Tumor Cells

BTLA expression on tumor cells was reported last year in lung cancer. Li et al. (40) found a positive BTLA expression in 35 on 87 lung adenocarcinoma biopsies. These 35 patients presented a shorter relapse-free survival compared to the BTLA negative group. BTLA expression on TILs was moderate. So far, BTLA tumor expression was assessed in one study. Feng et al. (41) described a positive BTLA expression on tumor cells in gastric cancer. Both studies on lung and gastric cancers assessed the BTLA expression by IHC with polyclonal anti-BTLA antibodies. Further analyses are required to explore the BTLA expression on non-immune cells. To note, BTLA upregulation have been reported in non-Hodgkin lymphoma (42). However, since BTLA is expressed by non-malignant B cells, the comparison with solid tumors is delicate.

## Discussion

Immuno-evasion through BTLA/HVEM was studied in many haematological malignancies and solid tumors. In lung cancer, data remain scarce but promising.

As we reviewed, HVEM can be overexpressed in tumor conditions. This upregulation is directly linked to tumor aggressiveness and correlated to a poorer prognosis. In lung cancer, Ren et al. (17) reported that HVEM expression was associated to disease progression, and was distinct from PD-L1 expression. However, in this study, HVEM expression was not correlated with the overall survival. In this cohort, 75% of patients were classified as early stage (I–II), moreover, molecular classification was not complete (only the EGFR and KRAS mutations status available). Finally, only 27 biopsies were HVEM positive in the advanced stage group (III–IV). Therefore, we hypothesize that HVEM is predictive of the OS only for a subset of patients that has yet to be defined. The small number of patients in the advanced-stage group is another limitation to assess the prognosis impact of HVEM for these patients. Further studies are required to evaluate the correlation between HVEM expression and clinical parameters in lung cancer.

Instead of PD-1, BTLA is only expressed on a small subset of T cells. Nonetheless, these BTLA^+^ lymphocytes, which are also PD-1^+^, TIM3^+^, CTLA-4^+^, and LAG-3^+^, seem to be terminally exhausted and poorly functional, and are associated to disease progression in lung cancer (38). These data are coherent with previous publications in other malignancies such as gastric cancer (11) or melanoma (35, 36). Recently, BTLA expression was described on tumor cells (40), raising questions about the role of BTLA on non-immune cells.

To conclude, BTLA-HVEM couple is truly involved in immune escape. Indeed, HVEM upregulation by tumor cells dampens anti-tumor immunity through BTLA engagement, resulting in disease progression and a poorer prognosis. The inhibitory signal triggered by BTLA can be overcome, as shown in melanoma (35, 36). In addition, combining blockade of BTLA and PD-1 has demonstrated an interesting synergistic effect. Therefore, targeting BTLA or HVEM represents a promising new IT that remains to be tested in lung cancer.

## Author Contributions

CD designed and wrote the review, and designed the figures. LG proofread and wrote the manuscript. DO provided the fundings and proofread the manuscript. All authors contributed to the article and approved the submitted version.

## Conflict of Interest

DO is a co-founder and shareholder of ImCheck Therapeutics, Alderaan Biotechnology and Emergence Therapeutics and has research funds from ImCheck Therapeutics, Alderaan Biotechnology, Cellectis and Emergence Therapeutics. The other authors declare no conflicts of interest. The funders had no role in the design of the study, in the writing of the manuscript, or in the decision to publish.

## Publisher’s Note

All claims expressed in this article are solely those of the authors and do not necessarily represent those of their affiliated organizations, or those of the publisher, the editors and the reviewers. Any product that may be evaluated in this article, or claim that may be made by its manufacturer, is not guaranteed or endorsed by the publisher.
